# Irritable Bowel Syndrome, Depression, and Neurodegeneration: A Bidirectional Communication from Gut to Brain

**DOI:** 10.3390/nu13093061

**Published:** 2021-08-31

**Authors:** Muhammad Nazirul Mubin Aziz, Jaya Kumar, Khairul Najmi Muhammad Nawawi, Raja Affendi Raja Ali, Norfilza M. Mokhtar

**Affiliations:** 1 Department of Physiology, Faculty of Medicine, Universiti Kebangsaan Malaysia, Kuala Lumpur 56000, Malaysia; muhammadnazirulmubin@gmail.com (M.N.M.A.); jayakumar@ukm.edu.my (J.K.); 2 Gut Research Group, Faculty of Medicine, Universiti Kebangsaan Malaysia, Kuala Lumpur 56000, Malaysia; khairulnajmi84@gmail.com (K.N.M.N.); draffendi@ppukm.ukm.edu.my (R.A.R.A.); 3 Gastroenterology Unit, Department of Medicine, Faculty of Medicine, Universiti Kebangsaan Malaysia, Kuala Lumpur 56000, Malaysia

**Keywords:** irritable bowel syndrome, microbiota-gut-immune-glia axis, depression, neurodegeneration, gut dysbiosis

## Abstract

Patients with irritable bowel syndrome (IBS) are increasingly presenting with a wide range of neuropsychiatric symptoms, such as deterioration in gastroenteric physiology, including visceral hypersensitivity, altered intestinal membrane permeability, and gastrointestinal motor dysfunction. Functional imaging of IBS patients has revealed several abnormalities in various brain regions, such as significant activation of amygdala, thinning of insular and anterior cingulate cortex, and increase in hypothalamic gray matter, which results in poor psychiatric and cognitive outcomes. Interrelations between the enteric and central events in IBS-related gastrointestinal, neurological, and psychiatric pathologies have compelled researchers to study the gut-brain axis—a bidirectional communication that maintains the homeostasis of the gastrointestinal and central nervous system with gut microbiota as the protagonist. Thus, it can be disrupted by any alteration owing to the gut dysbiosis or loss of diversity in microbial composition. Available evidence indicates that the use of probiotics as a part of a balanced diet is effective in the management of IBS and IBS-associated neurodegenerative and psychiatric comorbidities. In this review, we delineate the pathogenesis and complications of IBS from gastrointestinal and neuropsychiatric standpoints while also discussing the neurodegenerative events in enteric and central nervous systems of IBS patients and the therapeutic potential of gut microbiota-based therapy established on clinical and preclinical data.

## 1. Introduction

Irritable bowel syndrome (IBS) is a gastrointestinal-related disorder that manifests as persistent abdominal pain or discomfort, which is commonly correlated with altered bowel habits as well as defecation frequency and form [1,2]. Its global prevalence is estimated at 3−11% [1,3,4]. The risk of being diagnosed with IBS is marginally higher in women compared to men and in individuals aged ≤50 years [3,4]. Although IBS was once assumed to primarily affect the Western population, it is becoming increasingly prevalent in developing Asian countries, such as Malaysia (affecting 10.9−15.8% of the population), owing to the more widespread adoption of the Western lifestyle and diet [5,6,7]. The ethnic distribution in the incidence of IBS is estimated at 16.2−17.5%, 15.2−16.8%, and 10.9−15.8% among Chinese, Indians, and Malays, respectively [5,6,7].

IBS is frequently associated with pathophysiology, such as dietary sensitivity, inflammation, genetics, infection, visceral hypersensitivity, psychosocial distress, gut dysbiosis, and intestinal barrier deterioration (Table 1) [8,9]. These complex causative factors typically result in persistent abdominal discomfort and pain in IBS patients, which is severe enough to warrant a hospital visit. Based on Rome IV criteria and the Bristol Stool Form Scale (BSFS), IBS can be divided into several predominant subtypes, namely IBS-C (constipation), IBS-D (diarrhea), IBS-M (mixed type), and IBS-U (unclassified) [10,11]. This IBS classification process aids clinicians in determining the most optimal treatment strategies that are specifically tailored to the diagnosed predominant subtype [10,11].

Healthcare costs associated with IBS management including investigation, treatment, medication, and delivery are estimated at US $2 billion yearly in China, £45.6 to £200 million per year in the UK, and US $1562 to US $7547 per person yearly in the US [1,16,17]. The aforementioned figures signify the financial burden incurred by IBS, and some patients also lose their source of income due to the severity of IBS symptoms [1]. Such challenges can cause significant psychosocial distress, affecting social interactions, spontaneity, and freedom [1]. Occasionally, IBS patients also face stigma from physicians, family members, and colleagues, which further undermines their quality of life [1,18].

## 2. Irritable Bowel Syndrome and Gut Dysbiosis

The human gastrointestinal tract harbors a hundred trillion diverse and complex microbial communities, including bacteria, fungi, viruses, and archaea [19,20]. The gut microbiota plays an essential role in maintaining the host physiology, specifically related to metabolism, neuronal development, and immune response [21]. Human gut microbiota is divided into four major phyla, mainly the Firmicutes, Actinobacteria, Bacteroidetes, and Proteobacteria [19]. The Firmicutes phylum consists of hundreds of genera, including *Lactobacillus*, *Enterococcus*, *Bacillus*, *Ruminicoccus*, and *Clostridium* [22]. On the other hand, Actinobacteria phylum comprises of *Bifidobacterium* genus, and Bacteroidetes phylum is composed of *Prevotella* and *Bacteroides* genera [22]. Lastly, the Proteobacteria phylum comprises of few predominant genera, such as *Escherichia*, *Shigella*, and *Helicobacter* [22]. This highly heterogeneous microbial community is capable of rapidly adapting to environmental changes as well as host-derived stimuli such as diet, chemical exposure, and immunological response [19,21]. Disruptions to gut microbial composition, also known as gut dysbiosis, have been observed in several diseases, such as the functional gastrointestinal-related disorders [22].

Gut dysbiosis is considered as a one of the focal determinants in IBS etiopathogenesis [23]. Microbial populations, such as *Lactobacillus*, *Bifidobacterium*, and *Faecalibacterium*, are significantly depleted in IBS patients, with a profound influence on their health [24]. *Lactobacillus* is essential for elevating the mucin production in the intestinal lining, which in turn prevents the adherence of pathogenic microbes, such as bacteria (*Pseudomonas aeruginosa* and *Helicobacter pylori*), parasites (*Entamoeba histolytica*), and fungi (*Candida albicans*) [25]. Similarly, *Bifidobacterium* provides a mucosal barrier that is necessary in the overall maintenance of gut homeostasis [26]. *Faecalibacterium prausnitzii*, which serves as a major butyrate producer in the human intestine that is crucial for the maintenance of gut homeostasis, is often low in IBS patients [27]. The production of butyrate reduces the intestinal inflammation and releases other essential metabolites to enhance the mucosal barrier function [27].

In IBS patients, pathogenic microbial populations such as *Campylobacter jejuni*, *Campylobacter concisus*, *Clostridium difficile*, *Helicobacter pylori*, *Escherichia coli*, *Shigella* spp., and *Salmonella* spp. are also often enriched; thus, they can be considered as the risk factor for developing functional gastrointestinal disorders such as IBS [28]. For instance, *Campylobacter jejuni* and *Campylobacter concisus* are capable of disrupting the intestinal barrier by promoting cell death and increasing gut permeability [28]. The deterioration of this intestinal barrier following *Campylobacter* infection can be notably observed in the urinary lactose and mannitol (L:M) excretion ratio, with a significant increase even after 6 to 12 weeks of *Campylobacter* gastroenteritis [15]. However, the urinary L:M excretion ratio among post-infective IBS (PI-IBS) patients continued to increase, up to 4 years after initial infection [15]. Reportedly, during infection, also numerous immune-related cells particularly mast cells, macrophages, T lymphocytes, and several pro-inflammatory cytokines including TNF-α, IFN-γ, IL-3, IL-4, IL-5, and IL-6 were elevated. The elevation of these immune-related cells were normally associated with prominent effects on vascular permeability, motility, secretion, and pain signaling [14,15].

According to Dayananda and Wilcox, *Clostridium difficile* infection is more common in post-infectious IBS patients [29]. As *Clostridium difficile* releases toxins that are detrimental to the enteric glial cells, neurons, colonocytes, and enterocytes, it disrupts the gut homeostasis [28]. *Helicobacter pylori* is a Gram-negative bacterium normally found in the mucous epithelium of the gut [30]. This pathogenic microbial was recently reported to cause systemic inflammation, visceral hypersensitivity at the upper GI tract, and increased gut permeability [28,30].

Another pathogenic organism that is closely associated with IBS development is *Escherichia coli* virulent pathotype (diarrheagenic and adherent-invasive) [31]. The pathogenic *Escherichia coli* invades the intestinal barrier and causes gut hypersensitivity as well as inflammation [31]. Other pathogenic organisms frequently observed in IBS development are *Shigella* spp. and *Salmonella* spp., which recruit inflammation-related cells (lymphocytes, cytokines, macrophages, and mast cells), causing a severe immunological response, thereby increasing the intestinal permeability and gut hypersensitivity in IBS patients [28]. The relationship between gut dysbiosis and mental disorder such as depression has been intriguingly discussed in multiple studies for the past few years. Suggestively, gut microbials interact with the host via several routes, including neural, neuroimmune, and neuroendocrine pathways [32,33]. Few studies also indicated that a compromised intestinal barrier allows pathogenic microbial products to be translocated, thus modulating the central nervous system (CNS) function by heightening the immune response and through the hypothalamic–pituitary–adrenal (HPA) axis [34]. For instance, Jiang et al., 2015 reported that there was a significant abundance of Proteobacteria (*Pseudomonas aeruginosa, Pseudomonas putida,* and *Klebsielle pneumonia*), Bacteroidetes (*Alistipes*) populations, and markedly reduced Firmicutes population in patients with major depressive disorder, as compared to healthy individuals [34]. Purportedly, a notable increase of *Alistipes* can cause severe abdominal pain as well as gut inflammation among IBS patients. *Alistipes* is also capable of impeding the tryptophan availability and consequently disrupting the intestinal serotonergic process [34].

## 3. Irritable Bowel Syndrome and Depression

Clinical unipolar depression or major depression disorder is a common mental illness, affecting more than 300 million people worldwide [35]. Depression is a form of behavioral dysregulation caused by an interplay of many factors, such as the environment, gender, age, and comorbidity with other pre-existing illnesses, including IBS [36,37]. Patients with IBS often suffer from significantly higher levels of depression compared to healthy subjects [38,39,40,41] as well as individuals affected by inflammatory bowel disease (IBD) [42]. For example, according to the univariate analyses conducted by Midenfjord et al. (2019), IBS patients that suffer from psychological distress also report more severe gastrointestinal symptoms [43]. Lee et al. (2017) similarly found that severe depressive symptoms were associated with a high odds ratio for IBS [40]. Empirical evidence also indicates that psychosomatic symptoms, such as depression, result in a two-fold increase in the onset of gastrointestinal symptoms in IBS [44,45].

## 4. Cognition and Neurology in Irritable Bowel Syndrome

The association between IBS and cognitive function is rather inconclusive. However, as depression is strongly linked to cognitive deficit, and IBS patients are often depressed, they are hypothesized to suffer from some form of cognitive impairment. In line with this argument, some researchers have noted a reduction in verbal IQ (but no significant decline in performance on incidental memory assessment, troop color word test, or Wechsler Abbreviated Scale of Intelligence) in patients with IBS relative to their own performance IQ and compared with healthy controls [46]. Yet, other authors have failed to find a meaningful association between cognitive function (based on the Mini mental state examination, Trail-making tests, Grooved Pegboard test, Hopkins verbal learning test, brief visual memory test, Wechsler Adult Intelligence Scale 3rd Edition, Stroop test, or Controlled oral word association test) and IBS [47]. According to a more recent systematic literature review, there was insufficient evidence to show a relationship between IBS and cognitive deteriorations [48].

Nonetheless, the ample body of brain imaging findings points to the presence of differences between IBS patients and healthy controls, primarily in brain regions associated to stress [49], visceral stimulation [50], sensory integration [51,52], affective processing [53], cognitive/executive functions [51], and somatic pain [54]. Most of these findings indicate a greater engagement of regions associated with emotional processing, such as hypothalamus, amygdala, pregenual anterior cingulate cortex, and anterior insula [49,51,53,54,55,56,57], owing to the emotional component of pain and other associated symptoms of IBS, including anxiety and depression. Significant reductions in activity in the prefrontal cortex (dorsolateral prefrontal cortex and orbitofrontal cortex) and limbic areas, such as amygdala, hippocampus, and anterior cingulate cortex, have been identified in brains of depressed individuals [58,59,60]. The volumetric changes in the hippocampus are closely associated with the duration of depression and its episodes [61,62]. A meta-analysis of prior studies investigating the changes in brain activity in clinical depression during emotionally valanced tasks, cognitively demanding tasks, and resting conditions revealed altered common brain regions during the resting state and when engaged in cognitively undemanding tasks. According to Schmaal et al. (2017), patients suffering from major depressive disorder (MDD) also had thinner cortical gray matter in the orbitofrontal cortex, insula, anterior, and posterior cingulate, and temporal lobe, which were most pronounced during the first episode and in adult-onset MDD [63]. The authors also found evidence of regional reductions and a lower surface area of medial orbitofrontal cortex, superior frontal gyrus, somatosensory, motor areas, and higher-order visual areas in MDD patients [63].

## 5. Neurodegeneration in Irritable Bowel Syndrome: Roles of Enteric Nervous System

Neurodegenerative events in IBS are due to immune injuries in the submucosal and myenteric plexuses [64]. Neuronal injury affects the activation profile of enteric neurons, thus resulting in altered responses of submucous neurons [65]; secretory neurons of submucous plexus; and musculomotor neurons of myenteric plexus [66]. In a chronic and acute stress (CAS)-induced IBS rat model adopted by Li et al. (2016) in their study, accelerated transit of small intestine was accompanied by an increase in the secretory motor neurons in the submucosal plexus, along with an increase in the secretion of excitatory neurotransmitters of enteric nervous system, such as acetylcholine and vasoactive intestinal peptide (VIP) [66]. These findings are consistent with those previously reported by Palsson et al. (2004), who noted markedly escalated VIP levels in the intestinal plasma of IBS patients [67]. Apart from increased excitatory transmission, Li et al. (2016) also reported a reduced number of nitric oxide synthase (NOS)-positive inhibitory interneurons (nitrergic neurons), with no notable changes in total and cholinergic neurons in the myenteric plexus, suggesting that the loss of inhibitory and heightened excitatory enteric neurotransmission enhanced small intestinal motility in the CAS-induced IBS in rats [66].

Several authors also found that nitrergic neurons were also sensitive to the type of diet consumed. For example, in the study conducted by Ye et al. (2020), mice fed a Western diet exhibited delayed colonic transit and impaired electric field stimulation-induced colonic relaxation response due to an increase in myenteric neuronal pyroptosis, which is a novel form of programmed cell death [68]. The Western diet increased the expression of TLR4 and cleaved caspase-1 (marker of pyroptosis), which was accompanied by loss of myenteric nitrergic neurons, without affecting the population of cholinergic neurons, hence indicating the vulnerability of nitrergic neurons toward a high-fat diet. The same researchers also reported increased neuronal pyroptosis of myenteric neurons (mainly nitrergic neurons, but not cholinergic neurons) in colons of obese and overweight patients, corroborating the findings reported in animal studies [68]. According to Fan et al. (2018), the anti-enteric neuronal antibodies (AENA)-positive rate was higher in IBS patients than in patients with slow transit functional constipation and those with IBD, as well as healthy controls [69]. On the other hand, Pittock et al. (2011) failed to identify a significant difference in the AENA-positive rate between healthy subjects and individuals suffering from functional gastrointestinal diseases [70]. Fan et al. (2018) also reported that the exposure of moderately and highly AENA-positive sera from IBS patients to cultured myenteric neurons of Guinea pigs and human SH-Sy5Y cells led to neuronal apoptosis with a significant increase in the expression of anti-active caspase 3, TUNEL-positive cells, cleaved caspase 3, and pro-apoptotic factor Bax, and a decrease in inhibitor of apoptosis Bcl-2 [69].

## 6. Neurodegeneration in IBS: Roles of Central Nervous System

Patients with IBS are at a greater risk of developing neurodegenerative diseases, including Parkinson’s disease [71,72], and dementia [73]. Brain imaging studies have uncovered various abnormalities in brain regions of IBS patients, especially decreased functional connectivity density (FCD) in brain regions pertaining to emotional arousal, cognitive regulation, and afferent network, and increased FCD in regions associated with sensorimotor modulation [74]. Neurodegenerative features, such as white matter integrity loss, are increasingly being reported. For example, Fang et al. (2017) found a significant decrease in fractional anisotropy (FA; a measure of white matter integrity) and axial diffusivity (AD; density and diameter of axon) [75]. In their subsequent investigation, Fang et al. (2017) also observed increased mean diffusivity (MD; changes in myelin integrity or cell membrane) in the right retrolenticular area of the internal capsule, splenium of the corpus callosum (CC), right superior corona radiata, and right posterior of internal capsule of IBS patients [75]. Decreases in FA and AD values indicate that white matter integrity has been compromised due to axonal injury or loss, as detected by diffusion tensor imaging [76]. In addition to IBS, white matter damage in CC was also reported in other chronic pain-related studies, indicating the important role of CC in abnormal somatosensory or nociceptive processing in IBS [77].

White matter abnormalities in the retrolenticular area of internal capsule and corona radiata indicate disrupted ascending somatosensory and descending motor inputs [78]. Ellingson et al. (2013) also reported reduced FA values in the regions associated with sensory perception/integration and motor association/integration, such as thalamus and basal ganglia, and higher FA values in the CC and frontal lobe of IBS patients compared to healthy controls [56]. The discrepancies in the findings yielded by the aforementioned studies could be due to differences in sample size or the imaging methods used. It is worth noting that Hubbard et al. (2018) reported decreased FA in the right (but not left) dorsal cingulum in female adolescent IBS patients (*n* = 12; 11.96–18.5 years old) compared to controls (*n* = 12; 16.24 ± 1.89 years old), especially in the retrosplenial portion of the cingulum bundle [57]. On the other hand, they found no significant correlation between the changes in white matter abnormalities and disease duration, pain intensity, or psychometric measures. Using voxel-wise analysis of the diffusion parameters, Nan et al. (2018) found reduced FA and increased RD values in the genu of corpus callosum of female patients suffering from IBS-C (*n* = 20; 21.9 ± 1.41 years old) compared to healthy controls (*n* = 19; 22.74 ± 1.19 years old) [79]. The authors also reported greater white matter abnormalities in female patients with functional constipation (*n* = 18; 21.11 ± 1.28 years old) compared to those diagnosed with IBS-C. According to their analyses, the observed changes in FA values were negatively correlated with abdominal discomfort or pain intensity, whereas the RD values of CC were positively associated with abdominal discomfort or pain intensity, linking CC disorder with perception of pain [79]. Corpus callosum white matter abnormalities have also been reported in anxiety disorders [80], depression [81], bipolar disorder [82], mild cognitive impairment [83], and early course of cognitive decline in Alzheimer’s disease [84]. Corpus callosum is the largest tract connecting the left and right brain hemispheres, supplying the neural inputs for various regions involved in sensory and motor integration, cognition, and emotion [85,86]. Corpus callosum white matter abnormalities are the most consistently associated central degenerative feature in IBS, which appears to be due to altered pain perception in pediatric patients. However, whether these abnormalities are early signs of central nervous system (CNS) changes in IBS or a compensatory change in CNS due to altered bowel movement over time remains to be established.

## 7. Therapeutic Interventions in IBS: The Role of Antidepressants

The effectiveness of several antidepressant agents in IBS patients has been studied, including the tricyclic antidepressants (TCAs), selective serotonin reuptake inhibitors (SSRIs), and serotonin–norepinephrine reuptake inhibitors (SNRIs). The exact mechanism of action of antidepressants in IBS is presently not fully understood. Anxiolytic and antidepressant drugs may directly act on the enteric nervous system involving pain perception, visceral hypersensitivity, and gastrointestinal motility. Almost 90% of serotonin is produced by the enterochromaffin cells of the intestinal mucosa and has been shown to cause bloating, nausea, and vomiting [87]. According to Creed (2006), apart from their antidepressant effect in IBS patients with concomitant mood disorders, SSRIs might alter psychological processes, causing reduced somatization [88]. In line with this view, Kreiter et al. (2021) associated escitalopram treatment with changes in the symptom networks in IBS patients with panic disorder based on their electronic momentary assessments [89]. They also purported that an alleviation of physical symptoms was possibly due to healthier emotion regulation [89].

There are growing evidence of anti-inflammatory and anti-oxidative effects of antidepressant agents [90,91]. In the animal model of seizure by Sitges et al. (2014), the authors found that the administration of repeated dose of sertraline (SSRI) decreased the expression of IL-1β mRNA and TNF-αN in the hippocampus [92]. Another animal study by Rafiee et al. (2016) concluded the anti-inflammatory effect of fluvoxamine (SSRI). They had shown that the administration of fluvoxamine was able to significantly decrease the expression of inflammatory genes such as intercellular adhesion molecule (ICAM_1_), vascular cell adhesion molecule (VCAM_1_), cyclooxygenases2 (COX2), and inducible nitric oxide synthase (iNOS) [93]. Moreover, Venlafaxine (SNRI) was shown to have an inhibitory effect on superoxide generation by the microglia, albeit it had only a marginal effect on major pro-inflammatory parameters [94]. As the inflammation process has been postulated to play a role in the pathogenesis of IBS [95], it is wise to hypothesize that these antidepressant agents possibly alleviate the IBS symptoms through its anti-inflammatory and anti-oxidative effects.

TCAs are one of the oldest classes of antidepressants, and they have been used in IBS patients for more than four decades [96]. The most comprehensively studied TCAs include desipramine, trimipramine, imipramine, and amitriptyline. Evidence yielded by these investigations indicates that the dose employed in treating IBS is much lower (e.g., amitriptyline: 10−25 mg/day) compared to the therapeutic dose for depression (e.g., amitriptyline: 25−150 mg/day). SSRIs are among the newer classes of antidepressants, which include paroxetine, fluoxetine, escitalopram, and citalopram. As the name implies, SSRIs selectively inhibit serotonin reuptake into presynaptic cells by blocking the serotonin transporter. This leads to an abrupt increase in serotonin levels in the brain and eventually contributes to their therapeutic actions.

Ford et al. (2019) recently conducted a meta-analysis of 18 randomized controlled trials (RCTs) consisting of 11 TCA trials, 6 SSRI trials, and 1 trial involving both drug classes [97]. The pooled sample comprised of 1127 patients, 612 of whom received active therapy and 515 of whom received placebos. The proportion of patients with no IBS symptom improvement was much lower in subjects receiving TCAs compared to placebo groups (42.7%, 186/436 vs. 63.8%, 224/351, respectively) with the relative risk (RR) of 0.65 and number needed to treat (NNT) of 4.5. SSRIs also showed similar efficacy with the RR of 0.68 and NNT of 5 (seven trials, as a part of which 176 patients took SSRIs and 180 were given placebo) [97]. However, given the significant heterogeneity among studies and a broad range of 95% confidence intervals employed in analyses, the findings related to SSRIs have to be interpreted with caution. The authors of two small pilot non-randomized studies (*n* ≤ 15) examined the role of duloxetine (SNRI) in IBS patients. Their findings indicated a significant improvement in quality of life, abdominal pain, and anxiety; however, most participants experienced side effects such as fatigue, constipation, nausea, and insomnia [98,99].

For their more recent 6-week-long RCT of vortioxetine (SSRI), Seddighnia et al. (2020) recruited 72 patients and randomly assigned them to the vortioxetine (*n* = 36) and placebo (*n* = 36) groups [100]. The vortioxetine group demonstrated a greater increase in quality of life as compared to placebo (*p* < 0.01), irrespective of depression or anxiety score changes [100]. Vortioxetine is a potent 5-HT3 receptor antagonist, and suppression of the 5-HT3 receptor improves IBS symptoms such as abdominal pain, stool consistency, and gastrointestinal motility, as shown by studies involving 5-HT3 blockers (alosetron and ramosetron) [101,102]. In another recent RCT, Khalilian et al. (2018) examined the role of mirtazapine in the treatment of diarrhea-predominant IBS [103]. Mirtazapine is an atypical antidepressant agent with 5-HT3 receptor antagonist property. This 8-week trial involved 67 patients and showed promising results, including significant improvement in the severity of IBS symptoms (*p* = 0.002), quality of life (*p* = 0.04), and anxiety symptoms (*p* = 0.005). Most patients’ gastrointestinal symptoms (abdominal pain, urgency, and diarrhea) significantly improved, with the exception of bloating [103].

One of the major drawbacks of prescribing antidepressants to IBS patients is considerable side or adverse effects. According to Ford et al. (2019), the incidence of adverse effects was significantly higher among those taking antidepressants (RR = 1.56, NNT = 8.5) [97]. However, none of these adverse events was serious. Due to TCAs’ effect on muscarinic, alpha1 adrenergic, and histaminic receptors, side effects (such as drowsiness and dry mouth) are more common in patients taking TCAs as compared to SSRIs [97,100]. Given the shortcomings of extant studies, such as inadequate sample size, and inclusion of IBS patients with concomitant psychological disorders, as well as different subtypes of IBS [100], further investigations into the use of antidepressants in the management of IBS are warranted.

## 8. Therapeutic Intervention in IBS: Roles of Prebiotics, Probiotics, and Psychobiotics

Over more than a decade, studies in the literature have testified the alteration in the gut microbiota composition following diet modification for a long time [104]. Recently, the roles of pre- and probiotics are widened to include ecosystem regulation by modulating the immune system and exerting positive physiological effects as well as affecting the metabolic health of the host [105,106]. A prebiotic is a substrate that provides an optimal environment with minimal side effects for boosting the growth of beneficial gut microbiota. Normally, prebiotics are broken down by the anaerobic gut microbiota to produce fermentation products, including short-chain fatty acids (acetic acid, butyric acid, and propionate) and gases (carbon dioxide and hydrogen) [107]. Prebiotics are carbohydrate-based fibers that remain undigested in the human gut to support microbial survival. The most widely studied prebiotics are fructo-oligosaccharides and inulin [108]. The majority of prebiotics are taken orally at a daily dose of three to five grams. Natural sources of fructo-oligosaccharides are asparagus, wheat, garlic, and artichokes [109].

Low doses of functional food products containing prebiotics have a tendency to relieve IBS symptoms, including anxiety and depression [110]. Out of the four randomized controlled trials (RCTs) reported, only a single study involving 50 IBS patients showed improvement in global symptoms and bloating [110]. Following their meta-analysis involving 27 studies with a total of 2293 IBS patients of all subtypes, Lee et al. (2017) reported significantly higher levels of depression and anxiety in IBS patients as compared to healthy controls [40]. Further sub-analysis in which IBS subtypes were considered separately showed the highest depression among IBS-C patients [40]. More recently, our research team demonstrated that about 32.1% of the IBS-C patient population in Malaysia has sub-threshold or subclinical depression, which can be easily missed in clinical practice [37]. According to Bahrudin et al. (2020), the consumption of cultured drinks (probiotics) containing prebiotics in the form of polydextrose can significantly improve bowel function in IBS-C patients [111]. Polydextrose is a type of fermented soluble fiber, which is a low-calorie carbohydrate that can be added to dairy products and baked goods [111]. More RCTs involve the intake of either a single or a combination of prebiotics (inulin, galactooligosaccharides, and fructooligosaccharides) by patients suffering from a variety of acute and chronic diseases [106].

The acceptable definition of probiotics is “live microorganisms that give health benefits to the host when administered in adequate amount” (page 507) [112]. Probiotics were initially consumed by healthy individuals for maintaining health and reducing the risk of developing a disease, based on the belief that the consumption of probiotics would displace or replace harmful gut bacteria with beneficial microbiota [113]. However, over time, their use expanded to medicine, whereby they are now prescribed as a treatment modality or alternative therapy in multiple gastrointestinal disorders, including IBS [110]. Probiotics are administered in either a single or a combination of multiple strains of bacteria or fungi [114]. Commercially, probiotics are sold as foods (e.g., yogurt) and supplements (sachets). Lactobacilli and Bifidobacterium species are the most common ingredients of probiotics. The health benefits of probiotics were documented on the defined strain of gut microbiota. Among their actions include the production of antimicrobial agents, reduction of luminal pH, and displacement of pathobionts through competitive exclusion [113].

McCarthy et al. (2003) studied the effect of a probiotics murine model of colitis, whereby colitis was induced in an interleukin-10 (IL-10) knockout mouse model [115]. *Lactobacillus salivarius* UCC118 and *Bifidobacterium infantis* (*B. infantis*) 35,624 were chosen for their specific properties, including non-pathogenicity, ability to adhere to human epithelial cells, tolerance to intestinal acid and bile, and ability to survive in the human gastrointestinal tract [115]. The authors found that the administration of probiotics reduced mucosal inflammation, as indicated by declines in the levels of TNF-α, IFN-δ, and IL-12 in isolated splenocytes obtained from the knock-out mice, suggesting that probiotics ameliorate colitis through their actions on multiple pro-inflammatory mediators. The role of probiotics is likely to be effective in IBS, too, as there is increasing evidence of low-grade inflammation especially in post-infectious gastroenteritis [116].

The safety and efficacy of probiotics in IBS were tested in 16 double-blind placebo controlled RCTs as early as 1996, using the Manning criteria [117]. However, based on this systematic review, there was no single study that reported adverse events following probiotics consumption. It was difficult to come out with the final consensus on the use of probiotics in IBS because of the variability in the types and dosages of probiotics, the criteria used to define IBS subgroups, and small sample sizes [117]. Despite these limitations, findings yielded by the meta-analysis of single-center and multi-center studies with variable treatment durations (4−8 weeks) conducted by Yuan et al. (2017) indicated that a mixture of probiotics containing *B. infantis* 35,624 relieves IBS-related symptoms, including abdominal pain, distension, and change in bowel habits [118]. Therefore, the benefit of probiotics in IBS has a tendency to be symptom and strain-specific [110].

**Figure 1:**
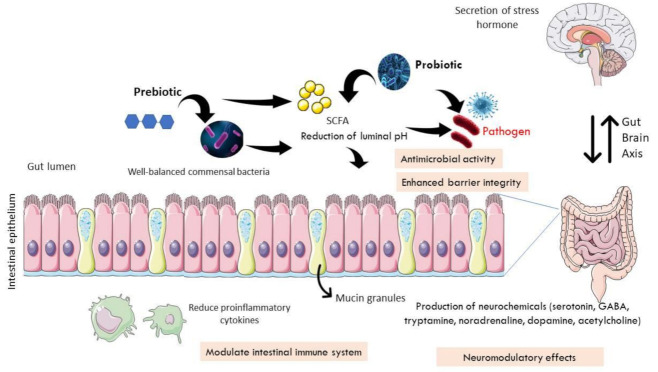
Mechanisms of prebiotic and probiotic action in modulating bidirectional gut−brain axis. A healthy human gut is unable to digest macronutrients, including plant-derived polysaccharides. Probiotics produce enzymes to digest the fibers and carbohydrates to produce short-chain fatty acids (SCFA) in the form of lactic acid and acetic acids. Prebiotics serve as a source of nutrition for the stimulation and propagation of commensal bacteria in the gut. The presence of SCFA reduces the pH of the intestinal lumen, preventing the growth of pathogen, or it has anti-microbial activity. Certain probiotic strains could restore the intestinal barrier function by increasing the expression of tight junction proteins as well as mucus-secretion genes. They also help modulate the intestinal immune system by reducing the amount of pro-inflammatory cytokines. In addition, probiotics exhibit neuromodulatory effects by enhancing neurochemical production in the gut, including serotonin, GABA, tryptamine, noradrenaline, dopamine, and acetylcholine. As cytokines and neurotransmitters will impair the integrity of the blood−brain barrier, this leads to potentially damaging effects of inflammatory or pathogenic elements that link to the central nervous system to secrete stress hormones.

Synbiotic is a combination of prebiotics and probiotics [107]. As an example of the use of synbiotics in IBS, a meta-analysis conducted by Ford et al. (2014) found two RCTs that were based in Italy and South Korea [107]. The single-blinded study in Italy used *L. acidophilus* and *L. helveticus*, with Bifidobacterium species, combined with phytoextract medium for 12-week duration. Meanwhile, the double-blinded RCT in South Korea utilized *B. lactis* with acacia fiber for 8-week. Both studies failed to demonstrate statistical significance in alleviating IBS symptoms. The authors attributed these results to the heterogeneity in dosage, duration, and combinations of prebiotics and probiotics used. Thus, they concluded that while probiotics provide good outcome for IBS patients, available evidence is insufficient to support the beneficial effects of either prebiotics or synbiotics. Intestinal microbiota is capable of producing neurochemicals that influence physiological processes in the brain and psychological symptoms via the microbiota–gut–immune–glia axis. Dinan et al. (2013) has introduced the term “psychobiotics”, referring to living microorganisms capable of producing neuroactive substances and providing benefits to the nervous system [119]. Their effectiveness was examined in a double-blind RCT using multiple bacterial species of *Lactobacillus acidophilus*, *L. plantarum*, *L. rhamnosus*, *Bifidobacterium breve*, *B. lactis*, *B. longum,* and *Streptococcus thermophilus* performed by Han et al. (2017) in 50 IBS-D patients based on Rome III criteria [120]. According to the authors, the supplementation of probiotics for four weeks was found to significantly improve depression symptoms, which were probably modulated by the gut−brain axis. The human gut microbiota promotes the biosynthesis of serotonin (5-hydroxytryptamine, 5-HT) in the enterochromaffin cells that secrete into lumen and modulate circulating serotonin [121]. In their analyses, the authors examined the effects of specific subsets of spore-forming bacteria, including *Bacteroides fragilis* and *B. uniformis*. They showed a schematic of prebiotics’ and probiotics’ roles in modulating neuroendocrine system in the enteric nervous system. Figure 1 shows the link of the enteric nervous system with the brain through the bidirectional gut−brain axis.

## 9. Conclusions

Irritable bowel syndrome is a functional gastrointestinal disorder that manifests as physical and mental symptoms. Gut dysbiosis is one of the fundamental theories that could explain this condition. Patients with severe IBS often suffer from psychological distress and report more severe gastrointestinal symptoms. Even though affective disorders such as depression and anxiety have been associated with IBS, such correlation is yet to be established from an empirical standpoint. Brain imaging studies of IBS patients indicate changes primarily in the areas related to pain and emotional processing, whereas the alterations in the depressed brains were more pertaining to learning and emotional regions, which corroborates the stronger association between depression and poor cognitive outcomes than IBS. Preclinical studies have related altered bowel movement in IBS to decreased inhibitory (nitregenic) and increased excitatory neurotransmission. The nitregenic neurons are sensitive to dietary intake, and undergo cell death in the colons of obese and overweight patients. However, in IBS, this is yet to be proven. The complex interactions between gut microbiota and the host’s nervous, immune, and endocrine system point to the favorable role of probiotics in managing IBS. Probiotics’ ability to alleviate the depressive symptoms associated with irritable bowel syndrome has demonstrated their therapeutic effect beyond the gastrointestinal tract via the gut−brain axis, which is a fascinating bidirectional pathway in humans.

## Figures and Tables

**Table 1:** Brief summary of pathophysiology that are known to cause irritable bowel syndrome.

Factors	Interpretation	References
Dietary Sensitivity	▪ Intake of fermentable oligosaccharides, disaccharides, monosaccharides, and polyols (FODMAPs)-related diet. ▪ Causes osmotic hypertension and excessive gases production.	Oświęcimska et al., 2017 [8]
Inflammation	▪ Elevate immune response in the enteric mucosa in IBS patients. ▪ Increase production of B cell, T cell, macrophage, and pro-inflammatory marker such as IL-6, IL-12, IL-8, IL-1ꞵ, and TNF-α.	Defrees and Bailey, 2017 [10]; Oświęcimska et al., 2017 [8]
Genetics	▪ 33% of diagnosed patients have IBS in their family history. ▪ Mutation in serotonin reuptake receptor (SERT) and sucrose isomaltase (SCN5A).	Black and Ford, 2020 [1]; Oświęcimska et al., 2017 [8]; Holtmann et al., 2016 [12]
Infection	▪ Reportedly, IBS will develop six-fold after infection with viral, bacteria, protozoan, and parasites. ▪ Examples of infection that can caused IBS: Bacteria (*Salmonella* spp., *Escherichia coli, Clostridioides difficile, Campylobacter jejuni, Vibrio cholera*) Virus (Norwalk viruses and noroviruses) Protozoan (*Trichinella* sp.) Parasite (*Giardia intestinalis*)	Black and Ford, 2020 [1]; Defrees and Bailey, 2017 [10]; Oświęcimska et al., 2017 [8]
Visceral Hypersensitivity (VH)	▪ 55% of IBS patients manifest this VH feature. ▪ VH can be referred to as enhanced pain and nociceptive sensation when there are bowel contractions and distensions. ▪ One of the key hallmarks in IBS symptoms.	Defrees and Bailey, 2017 [10]; Farzaei et al., 2016 [13]
Increased Intestinal Permeability	▪ Serve as a protective fence between intraluminal contents (food, microflora, antigens, toxins, and ingested bacteria) and the body, penetration of these materials can stimulate immunological response and causes luminal inflammation. ▪ This condition was frequently observed in IBS patients regardless of the predominant subtypes.	González-Castro et al., 2017 [14]; Oświęcimska et al., 2017 [8]; Camilleri et al., 2012 [15]
Gut Dysbiosis	▪ Evidently, gut dysbiosis occurrence was closely associated with IBS incident. ▪ For instance, depletion of beneficial microorganism (*Bifidobacterium* and *Lactobacillus* spp.) with increased growth of pathogenic microorganism (*Veillonella, Haemophilus, parainfluenzae, Enterobacter,* and *Streptococcus* spp.) can be observed in IBS patients.	Hadjivasilis et al., 2019 [9]; Oświęcimska et al., 2017 [8]
Psychosocial Distress	▪ 75% of IBS patients were associated with psychosocial comorbidity. ▪ Approximately 30% to 50% of IBS patients suffer from anxiety and hopelessness. ▪ Around 30% of IBS patients suffer from mood disorder. ▪ Approximately 15% to 30% of IBS patients also experienced suicidal thought. ▪ Somatization occurrence was also prevalent in the psychosocial distress of IBS patients.	Black and Ford, 2020 [1]; Hadjivasilis et al., 2019 [9]
